# Protein Expression Status of *HTR4* and *PDE4D* Genes in Glial Brain Tumors Followed by the Study of Genomic Instability

**DOI:** 10.3390/life16030374

**Published:** 2026-02-25

**Authors:** Marina Milić, Nejla Ademović, Emilija Manojlović Gačić, Vladimir Baščarević, Nasta Tanić, Nikola Tanić, Ivan Milić

**Affiliations:** 1 Clinic for Neurosurgery, University Clinical Center of Serbia, Faculty of Medicine, University of Belgrade, 11000 Belgrade, Serbia; dr.marina.r@gmail.com (M.M.); vladabascarevic@gmail.com (V.B.); 2 Department of Neurobiology, Institute for Biological Research “Siniša Stanković”, National Institute of Republic of Serbia, University of Belgrade, 11000 Belgrade, Serbia; nejla.ademovic@ibiss.bg.ac.rs (N.A.); nikolata@ibiss.bg.ac.rs (N.T.); 3 Institute of Pathology, Faculty of Medicine, University of Belgrade, 11000 Belgrade, Serbia; emanojlovicgacic@gmail.com; 4 Department of Radiobiology and Molecular Genetics, Institute of Nuclear Sciences “Vinča”, National Institute of Republic of Serbia, University of Belgrade, 11000 Belgrade, Serbia; nastad@vin.bg.ac.rs

**Keywords:** glioma, 5-HTR4, PDE4D, genomic instability (GI), microsatellite instability (MIN), chromosomal instability (CIN)

## Abstract

Malignant gliomas are the most common primary tumors of the central nervous system (CNS), originating from glial cells. They account for 30% of all CNS tumors. Among them, glioblastoma (GBM) is the most common, accounting for 45% of all glial tumors, while low-grade gliomas (LGGs) account for 31.8% of all gliomas. The aim of this study was to analyze the protein-expression profile of *HTR4* and *PDE4D* genes in patients with glioma by immunohistochemical (IHC) analysis, to determine whether some interrelationship between them exists, to correlate their expression with clinical and histopathological parameters and therapy, and to determine their impact on patients’ survival. In addition, we analyzed the level of genomic instability (GI) (microsatellite (MIN), chromosomal (CIN) and total GI) by AP-PCR, in order to understand whether it can represent a tool for biological stratification of glioma tumors and risk assessment. Our results revealed that synchronized expression of 5-HTR4 and PDE4D proteins represents a stable modulatory signaling axis of glial-tumor biology, and reflects the activity of cAMP signaling pathway, but cannot independently stratify patients. Moreover, our study confirms that the combination of MIN, CIN and total GI represents a powerful tool for biological tumor stratification, risk assessment and understanding the pathobiological spectrum of the disease.

## 1. Introduction

Malignant gliomas are the most common primary tumors of the central nervous system (CNS), originating from glial cells. They account for 30% of all CNS tumors [1]. Among them, glioblastoma (GBM) is the most common, accounting for 45% of all glial tumors [2], while low-grade gliomas (LGGs) account for 31.8% of all gliomas [3]. Previous WHO classification of tumors of the CNS from 2016 classified diffuse gliomas on the basis of morphological features. The latest WHO classification from 2021 [4] emphasizes the role of molecular features, and classifies them into adult-type (astrocytoma, IDH-mutant, oligodendroglioma (ODG), IDH-mutant, and 1p/19q-codeletion and GBM, IDH-wild type) and pediatric-type (LGGs and high-grade gliomas (HGGs)). Circumscribed astrocytic gliomas (CAGs) include pilocytic astrocytoma (PCA), high-grade astrocytoma with piloid features (HGAP), pleomorphic xanthoastrocytoma (PXA), subependymal giant cell astrocytoma, chordoid glioma and astroblastoma, *MN1*-altered [4]. Glial tumors also include ependymomas and choroid plexus tumors. According to the appearance of the cells, the growth potential and the possibility of expansion, they are classified into four grades. LGGs include grade 1 and 2, and HGGs include grade 3 and 4.

Cancer, in general, is primarily a genetic disease, characterized by the alterations in deoxyribonucleic acid (DNA) sequences, copy number aberrations, chromosomal rearrangements and alterations in DNA methylation pattern. All this leads to disruption of homeostasis and cell proliferation. Studies carried out over the past three decades suggest that malignant gliomas, like other cancers, represent a consequence of the accumulation of genetic alterations. Their nature and exact number required for promotion and progression of malignant tumors still remain unclear. Spontaneous mutation rates in human cells are considerably lower than in cancer cells, therefore cancer cells must be a manifestation of the mutator phenotype. The mutator phenotype is also known as genomic instability (GI) and designates the increased mutation rate that occurs in neoplastic cells. The induction of the GI phenotype is a crucial early event in carcinogenesis. It enables an initiated cell to evolve into a cancer cell, by achieving a greater proliferative capacity and genetic plasticity, which can overcome host immunological resistance, local toxic environment and a suboptimal supply of micronutrients [5,6,7]. GI plays an important role, not only in tumor formation, but in progression, metastasis and resistance to chemotherapy (CTx) and radiotherapy (RT), and is one of the main mechanisms of heterogeneity within tumors [8,9,10,11]. There are three types of GI: chromosomal instability (CIN), microsatellite instability (MIN or MSI) and single nucleotide instability (SNI). CIN is characterized by changes in the number and structure of chromosomes [12], and MIN by changes in the number of oligonucleotide repeats in microsatellite regions, mainly caused by the loss of function of the mismatch repair (MMR) system [13,14,15,16].

However, very little information on GI in gliomas of different types and grades is available. In this study, we used arbitrarily primed polymerase chain reaction (AP-PCR) to detect and measure GI in 104 patients with glioma of different types and grades (Table 1). This is a follow-up study [17,18] on a different/new cohort, with more types of glial tumors included. Genomic instability designates the increased mutation rate all over the genome, genetic and epigenetic, which can affect a wide range of genes. Our previous results, mentioned above, identified alterations in some genes never before described in patients with glioma, among which were *HTR4* and *PDE4D.* Our assumption was that mutations accumulated in both, the *HTR4* and *PDE4D* genes, are the consequences of GI. As GI affects expression of genes, as well as the regulation of expression, we decided to analyze protein expression of these two.

*HTR* genes encode 5-hydroxytryptamine (serotonine) receptors (5-HTR) that include seven families. 5-HTR4 are coupled to a stimulatory Gs protein, which, when serotonin binds to the receptor, activates adenylyl cyclase (AC). This enzyme converts adenosine triphosphate (ATP) to 3′-5′-cyclic adenosine monophosphate (cAMP), increasing the cAMP level. 5-HTR4 are expressed in the CNS and peripheral nervous system. Apart from normal cells, cancer cells also synthesize and release serotonin and express 5-HTRs [19]. Serotonin affects cancer progression directly and indirectly by modulating the immune cells [20,21]. Serotonin and serotonin receptors have a dual role in the development of cancer. They can act as oncogenes and tumor suppressor genes (TSGs). On the one hand, they can stimulate the formation and progression of tumors, and on the other, they can lead to programmed cell death [22].

*PDE* genes encode phosphodiesterases (PDEs), enzymes that play a role in intracellular signal transduction. The *PDE4D* gene encodes cAMP-specific 3′,5′-cyclic phosphodiesterase 4D (PDE4D), which hydrolyzes cAMP and degrades it. Depending on the type of tumor, its grade and microenvironment, this enzyme can play a role in the formation and progression of tumors and have an antiapoptotic effect, but it can also have an antitumorigenic effect. It acts as both an oncogene and TSG. Studies have demonstrated that increased expression of the *PDE4D* gene, and consequently lower level of cAMP, correlate with a shorter survival time of patients with different types of tumors [23,24,25].

“Coordinated expression of these two proteins may reflect tumor biology, due to their opposite biochemical roles in cAMP metabolism, which regulates various physiological and pathological processes, like cell cycle, apoptosis, growth, differentiation, metabolism, migration, invasion and metastases. Also, cAMP regulates neurotransmitter synthesis and immune cell function. It achieves this through its effectors: protein kinase A (PKA), exchange protein activated by cAMP (EPAC) and ion channels independent of phosphorylation. PKA phosphorylates various proteins—transcription factors (CRE-binding protein (CREB), cAMP-responsive modulator (CREM) and activating transcription factor 1 (ATF1), which regulate the expression and transcription of genes, oncogenes and TSGs. Exchange protein activated by cAMP is a guanine nucleotide exchange factor, that in the presence of cAMP activates GTPase dependent enzymes (Ras-associated protein 1 (Rap1) and Ras-associated protein 2 (Rap2)). All this can affect tumor growth, depending on the tumor type, its environment and context [26,27]”.

The aim of this study was to analyze the protein-expression profile of HTR4 and PDE4D genes in patients with glioma, to determine whether some interrelationship between them exists, to correlate their expression with demographic, clinical and histopathological parameters and therapy, and to determine their impact on patients’ survival. In addition, we analyzed the level of GI (MIN, CIN, total GI), in order to understand whether it can represent a tool for the biological stratification of glial tumors and risk assessment.

## 2. Materials and Methods

### 2.1. Patients

The research was conducted as a prospective, experimental study of patients with histologically confirmed glial tumors of the CNS. Brain tumor tissues and blood samples were collected from 104 patients who underwent surgery at the Clinic for Neurosurgery of the University Clinical Center of Serbia in 2021. The tumor type (glial tumor) was proven by histopathological analysis (Table 1). The classification of these CNS tumors was performed according to WHO criteria from 2021 [4]. Tumor tissue samples were frozen in liquid nitrogen immediately after surgery, and then transferred to a freezer at −80 °C, where they were stored until the DNA-isolation procedure. From each patient, 3 mL of blood was taken from the cubital vein, and whole blood with anticoagulant was stored in a freezer at −20 °C, until the DNA-isolation procedure. Due to the impossibility of taking healthy brain tissue from all patients, in addition to the tumor tissue, which we needed for RT-PCR, because of the location of the tumor in or near the eloquent areas of the brain, we did not study mRNA expression of the *HTR4* and *PDE4D* genes. Samples were collected and analyzed after obtaining permission from the Ethics Committee of University Clinical Center of Serbia, the Ethics Commission of the Faculty of Medicine of the University of Belgrade and the written consent of patients or their guardians, in accordance with the ethical standards of the Declaration of Helsinki adopted in 1964 by the World Medical Association. DNA isolation was performed at the Institute for Biological Research “Siniša Stanković”, and immunohistochemical (IHC) analysis at the Institute of Pathology, Faculty of Medicine, University of Belgrade. Patients were monitored from the day of surgery until death or the last control.

### 2.2. DNA Fingerprinting (AP-PCR)

Genomic DNA was isolated from tumor tissue according to the protocol described by Sambrook et al. 1989 [28], while genomic DNA was isolated from blood according to the procedure described by Kunkel et al. 1977 [29]. Quality control of the isolated DNA was performed using agarose gel electrophoresis method. DNA profiling of tumors and blood was performed using AP-PCR. Optimization of AP-PCR reaction conditions was done for four tested primers (Mycrosynth, Balgach, CH) (carbonic anhydrase 12 forward (CA12F), carbonic anhydrase 9 reverse (CA9R), multi-drug resistance (MDR2) and cellular myelocytomatosis oncogene (C-MYC1) according to the modification by Cobb in 1997 [30], using Taguchi and Wu, 1980 model [31]. Primer sequences, AP-PCR reaction conditions and the composition of the reaction mixtures are shown in Table 2.

**Figure 1:**
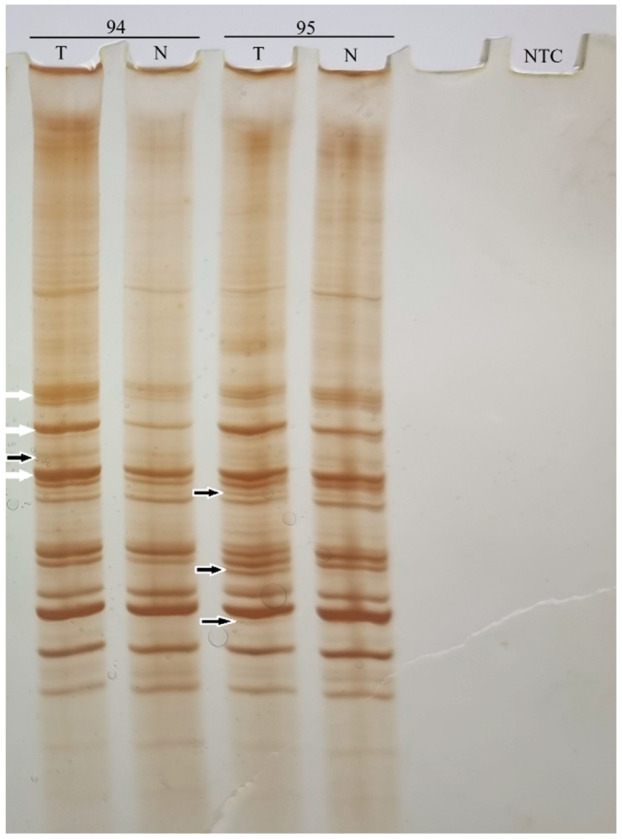
AP-PCR DNA profiles of glial tumor (T) and normal tissue (blood) (N) obtained with Ca12F primer. The numbers 94 and 95 indicate the serial number of the patients. Black arrows point to MIN and white arrows point to CIN; NTC—no-template control.

Pairs of DNA profiles of tumor tissue (glioma) and healthy tissue (blood) from each individual patient were analyzed, to detect differences in the number and intensity of bands. Qualitative changes are detected as changes in the layout and number of bands, i.e., as their presence or absence in the tumor tissue (glioma), in relation to the control healthy tissue (blood). They are an indicator of MIN. Quantitative changes are detected as differences in the intensity of bands of the same length in DNA profiles of tumor (glioma) and control healthy tissue (blood). They are an indicator of CIN. The characteristic appearance of DNA profiles of tumor and normal tissue obtained by AP-PCR is shown in Figure 1. Frequency of qualitative DNA changes (MIN) and quantitative DNA changes (CIN) were calculated as the number of qualitative (quantitative) DNA changes in tumor sample/total number of bands in healthy control tissue (blood) from the same patient. Frequency of total DNA changes (GI) was calculated as number of differential bands in tumor tissue/total number of bands in healthy control tissue (blood) of the same patient (MIN +CIN).

### 2.3. Immunohistochemistry

IHC analysis was performed on selected formalin-fixed paraffin-embedded (FFPE) tissue samples of tumor parenchyma, and mounted on silanized glass slides. The samples were deparaffinized, rehydrated and subjected to antigen retrieval in citrate puffer (pH 6), followed by blocking of endogenous peroxidase and nonspecific binding. Then sections were incubated with the primary antibodies: 5-HTR4 polyclonal rabbit antibody NBP2-30882 (Novus Biologicals, Centennial, CO, USA) (dilution 1:100, detection system: UltraVision Quanto HRP, Epredia, Portsmouth, NH, USA) and PDE4D polyclonal rabbit antibody NBP2-34500 (Novus Biologicals, Centennial, CO, USA) (dilution 1:200, detection system: UltraVision Quanto HRP, Epredia, Portsmouth, NH, USA), according to the manufacturer’s instructions. Control tissue sections included placenta tissue for 5-HTR4, and kidney tissue for PDE4D. Visualization was performed by standard diaminobenzidine (DAB) method.

### 2.4. Immunohistochemical Evaluation

The Immunoreactive Score (IRS) was used to assess the level of 5-HTR4 expression [32]. IRS is calculated by multiplying staining intensity (SI) (0–3) by the percentage of positive cells (PP) (0–4) (Table 3). The interpretation of IRS is shown in Table 4.

When it comes to PDE4D, SI was evaluated as negative (0) and positive: weak (1), moderate (2) and strong (3).

### 2.5. Statistical Analysis

For each patient, the following were recorded: sex, age, smoking status, tumor type, tumor category (LGG/HGG), tumor grade, application of RT and CTx, follow-up time and outcome status (event/censored). MIN total, CIN total and total GI were determined, as well as the protein expression profile of the *HTR4* and *PDE4D* genes. For each biomarker (MIN total, CIN total and total GI) the raw, mean, median and cluster values (obtained by algorithmic classification, cluster analysis based on distribution limits) [33] were calculated, whereby patients were classified into Lower and Upper groups according to the natural separation of data.

Statistical analysis was performed in several steps to evaluate the differences between groups, the correlation of biomarkers and the relationship of biomarkers with survival outcome. The non-parametric Mann–Whitney U test was used to compare continuous parameters between binary groups. For the comparison of categorical variables, the following were applied: χ^2^ test, Fisher’s exact test with presentation of Pearson χ^2^, *p*-values, odds ratio (OR) with 95% confidence intervals. To determine the association of biomarkers with overall survival, Kaplan–Meier analysis was applied, with Log Rank, Breslow, and Tarone–Ware tests. Statistical analyses were performed using validated, standard biostatistical software packages, specifically IBM SPSS Statistics (Version 27), R (version 4.x), and Statistica (Armonk, NY, USA). These platforms were used for cluster-based stratification of biomarkers. The level of statistical significance was set to *p* < 0.05 for significant findings and *p* = 0.05–0.10 for borderline/significant-informative trends. The application of parallel divisions of biomarkers (mean, median, cluster), combined with non-parametric and categorical tests, enabled a precise, multi-level assessment of their biological informativeness.

## 3. Results

### 3.1. Tissue Samples

Our study included 104 patients with glial brain tumors that were surgically removed and histopathologically and molecularly identified, according to the classification of tumors of CNS issued by the WHO in 2021 [4]. Patient’s characteristics are shown in Table 5.

### 3.2. Protein Expression of HTR4 and PDE4D

**Figure 2:**
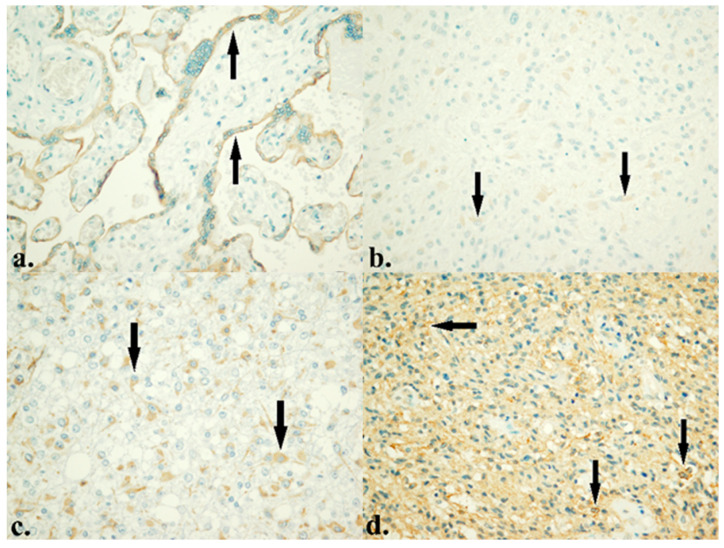
IHC expression of 5-HTR4 protein. (**a**) Expression of 5-HTR4 in placenta (positive control). (**b**) Low expression of 5-HTR4 in GBM. (**c**) Moderate expression of 5-HTR4 in ODG, CNS WHO grade 2. (**d**) High expression of 5-HTR4 in astrocytoma, CNS WHO grade 4 (×400).

**Figure 3:**
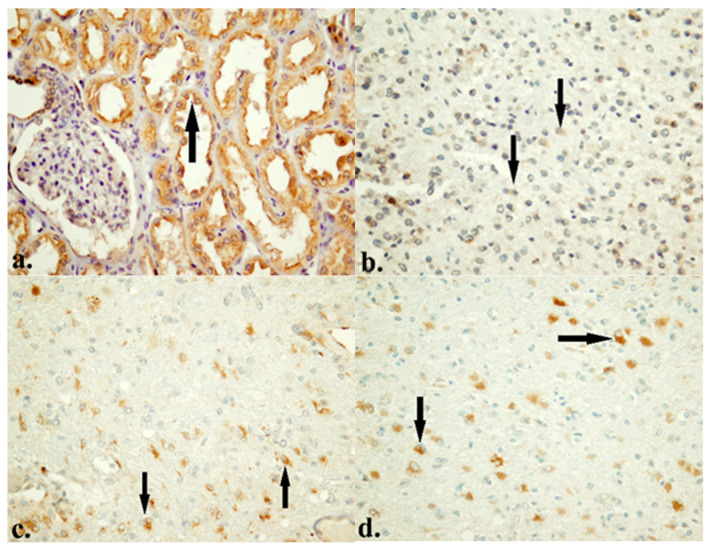
IHC expression of PDE4D protein. (**a**) Expression of PDE4D in kidney (positive control). (**b**) Low expression of PDE4D in GBM. (**c**) Moderate expression of PDE4D in astrocytoma, CNS WHO grade 3. (**d**) High expression of PDE4D in GBM (×400).

The results of IHC analysis of protein expression showed that levels of 5-HTR4 and PDE4D varied significantly among patients. Namely, 83.7% of patients showed low levels of 5-HTR4 expression, while 16.3% had high levels of 5-HTR4 expression (Figure 2). Similarly, 85.6% of these patients showed low levels of PDE4D expression, and 14.4% had high levels of PDE4D expression (Figure 3).

Patients were divided into two groups, according to IRS, which was used to evaluate the protein-expression status of the *HTR4* gene (Low with IRS of 0–3, N = 87 and High with IRS of 4–12, N = 17).

For the analysis of biological and prognostic significance of the protein PDE4D, the samples were divided into two groups according to SI (Low with SI score of 0–1, N = 89 and High with SI score of 2–3, N = 15).

**Figure 4:**
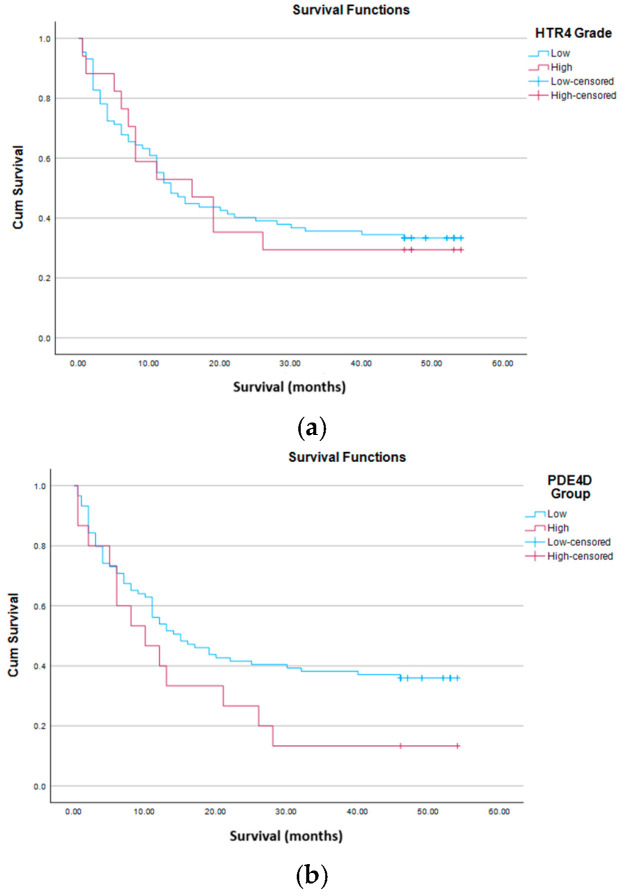

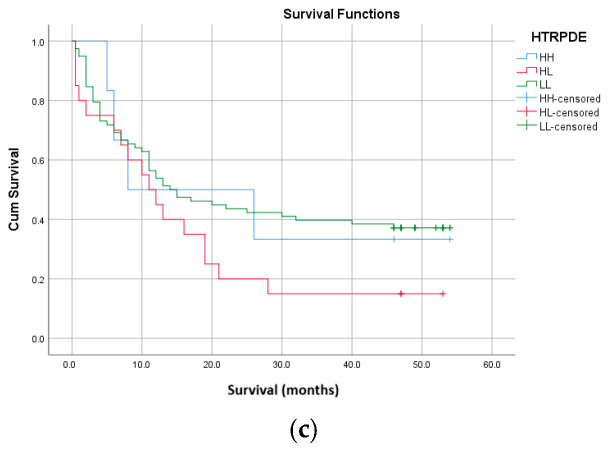
(**a**) Kaplan–Meier curves for 5-HTR4 protein. Log Rank (*p* = 0.871), Breslow (*p* = 0.954), Tarone–Ware (*p* = 0.959). (**b**) Kaplan–Meier curves for PDE4D protein. Log Rank (*p* = 0.096), Breslow (*p* = 0.191), Tarone–Ware (*p* = 0.139). (**c**) Kaplan–Meier curves for 5-HTR4/PDE4D proteins co-expression. Log Rank (*p* = 0.201), Breslow (*p* = 0.345), Tarone–Ware (*p* = 0.273).

The IHC analysis of 5-HTR4 clearly highlighted the predominant low protein expression in this cohort. The most important result of the entire IHC analysis is the strong association between 5-HTR4 and PDE4D (Table 6). In the HTR4 high group, PDE4D high is 35.3%, and in the HTR4 low group, PDE4D high is 10.3%. χ^2^ = 7.17, *p* = 0.007, OR = 4.73 (95% CI 1.41–15.86). This means that patients with high HTR4 are about 4–5 times more likely to be in the PDE4D high group. Analyzing the effects of these proteins individually, we did not get any statistically significant association with clinical or histopathological parameters, except that PDE4D high has a worse outcome, but only in numerical terms. Survival analyses did not reveal significant results for each individual protein (Figure 4a,b), so they cannot be considered as independent prognostic markers. When we analyze them as a co-expressed package, a trend is obvious, although still without statistical significance (Figure 4c).

Multivariate regression was intentionally not performed for the following methodological reasons:

(a) Limited number of “high expression” cases: HTR4 high: 17 patients (16.3%), PDE4D high: 15 patients (14.4%). Multivariate logistic or Cox regression requires an adequate number of events per variable (EPV ≥ 10 is standard). With such small subgroup sizes, a multivariate model would be statistically unstable, produce unreliable hazard ratios, risk overfitting and inflate standard errors. Thus, performing multivariate modeling would not provide valid or reproducible estimates.

(b) No significant univariate signal: 5-HTR4 and PDE4D individually were not significantly associated with major clinicopathological parameters or survival. In standard statistical modeling practice, multivariate modeling is justified when there is evidence of univariate association or strong biological confounding. Since no strong univariate predictors were identified for these proteins individually, multivariate adjustment would not meaningfully improve inference.

(c) Biological focus of the study: the primary objective of this study was to explore signaling-axis behavior (5-HTR4–PDE4D co-expression), to evaluate genomic instability markers (MIN total, CIN total, Total GI) and to assess stratification potential. The only biomarker demonstrating stable prognostic behavior was Total GI, not individual protein expression. Therefore, emphasis was placed on biologically coherent stratification rather than forced multivariable adjustment. Given the relatively small size of the high-expression subgroups and the absence of strong univariate signals, multivariate regression would risk overfitting and unstable parameter estimation. Therefore, we intentionally avoided presenting statistically unreliable multivariable models.

### 3.3. Analysis of Genomic Instability in Glioma

We analyzed GI in 104 patients with glial brain tumors (Table 1) and corresponding control samples (blood of corresponding patients). For that purpose, we calculated the frequency of qualitative (MIN) and quantitative (CIN) DNA changes, by dividing the number of altered bands in the AP-PCR profile of tumor tissue by the total number of bands in the control tissue of the same patient. MIN (MIN total mean 0.18057) and CIN (CIN total mean 0.17619) equally contributed to the GI of brain tumors in our cohort.

Analysis of MIN total (mean, cluster and median) showed that tumor category (LGG vs. HGG) was the only histological finding of significance (χ^2^ = 4.140, *p* = 0.042, OR = 0.367 (CI 0.137–0.984). In upper MIN total cluster and MIN total mean, MIN total was significantly higher in LGG than in HGG. OR 0.367 means that tumors with lower MIN total cluster and mean are almost three times more likely to be HGG.

**Figure 5:**
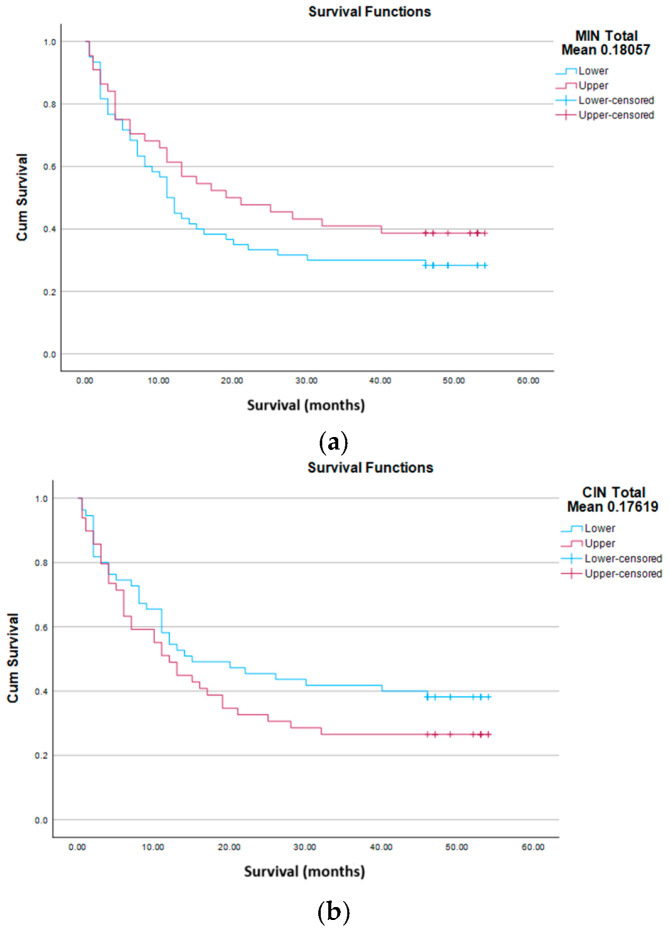

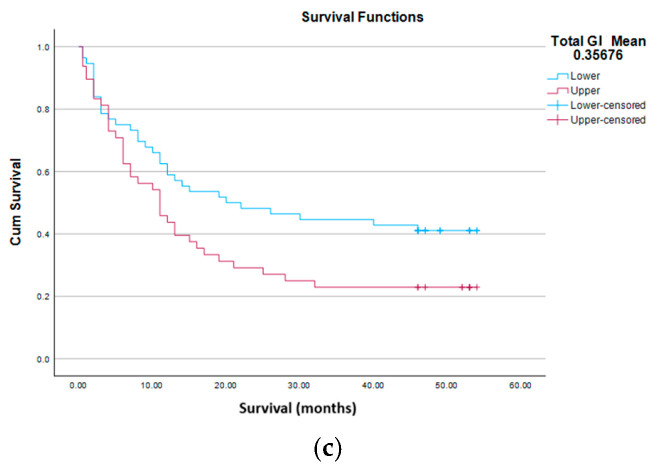
(**a**) Kaplan–Meier curves for MIN total mean. Log Rank (*p* = 0.227), Breslow (*p* = 0.250), Tarone-Ware (*p* = 0.230). (**b**) Kaplan–Meier curves for CIN total mean. Long Rank (*p* = 0.203), Breslow (*p* = 0.271), Tarone–Ware (*p* = 0.231). (**c**) Kaplan–Meier curves total GI mean. Log Rank (*p* = 0.045), Breslow (*p* = 0.078), Tarone–Ware (*p* = 0.057).

Kaplan–Meier analysis showed no association of MIN total (cluster, mean, median) with overall survival, as shown in the graph at Figure 5a.

Analysis of CIN total (mean, cluster and median) showed similar results. CIN total was independent of sex, smoking status, type, grade and category of tumor, application of CTx and RT, expression status of *HTR4* and *PDE4D* genes, status (event vs. censored) and had no effect on survival, as shown by the Kaplan–Meier curves (Figure 5b).

However, we got an interesting result when we analyzed the relationship between MIN total (mean, cluster and median) and CIN total (mean, cluster and median). Namely, MIN total follows CIN total stably, but with moderate intensity, χ^2^ = 3.939–6.522, *p* = 0.011–0.047, and OR = 2.224–2.788. CIN total mean and median parameters are statistically significantly higher in the upper MIN total group (cluster, mean and median). CIN total cluster represents the trend in the case of MIN total cluster and mean, but in the case of MIN total median there is statistical significance (Table 7).

Total GI was the sum of MIN total and CIN total. In the case of total GI cluster, at the cut-off for total GI mean and median, absolute separation occurs: total GI mean: χ^2^ = 60.387, *p* < 0.001; total GI median: χ^2^ = 70.452, *p* < 0.001. Therefore, MIN total, CIN total and total GI clearly separate the tumor profile in terms of the level of GI. Total GI is the most stable and statistically powerful biomarker of our cohort. Cluster separation is almost perfect, because there is no overlap. Total GI high group consistently has higher MIN total, higher CIN total, more events and shorter survival. The MIN total marker shows a very strong association with CIN total and total GI. CIN total also shows a strong association with MIN total and total GI. Regarding the optimal method of division, cluster analysis is by far the most powerful, median is reliable and stable, and mean is the least precise.

Table 8 shows that male sex was more common in the lower total GI cluster group, and female in the upper (χ^2^ = 4.062, *p* = 0.044, OR = 2.261). HTR4 high was more represented in the upper total GI cluster group (χ^2^ = 4.364, *p* = 0.037, OR = 3.792). Total GI (mean, cluster and median) is independent of smoking status, type, grade and category of tumor, application of CTx and RT and expression status of *PDE4D* gene. There is a statistically significant difference in the status (censored vs. event): in the upper total GI mean group there are significantly more events 37 (77.1%), than in the lower 33 (58.9%), χ^2^ = 3.871, *p* = 0.049, OR = 2.344 (Table 8). This means that a higher total GI mean is associated with a higher probability of a patient experiencing an outcome, event during follow-up. Kaplan–Meier curves (Figure 5c) confirm worse outcome in the upper total GI mean group.

## 4. Discussion

The primary aim of our study was to analyze the protein expression profile of *HTR4* and *PDE4D* genes in patients with different types of glioma and, in addition, to analyze GI in these tumors. Gliomas are the most common primary tumors of CNS, and among them GBM is the most aggressive and the most common. We were driven by the results that we obtained in our previous study, on different sample cohort [18], where we discovered mutations in these two genes. Both genes/proteins take part in the cAMP signaling pathway with opposite roles. 5-HTR4 increases cAMP levels [34], while PDE4D decreases them [35]. The regulation of the intracellular level of cAMP is very important for the formation and progression of glial tumors, and it would be expected that these two genes have the opposite expression.

However, one of the most significant and the most surprising results of our study is the strong association of 5-HTR4 and PDE4D expression. Namely, patients with high expression of 5-HTR4 have high expression of PDE4D and vice versa, those with low expression of 5-HTR4 have low expression of PDE4D. This result acknowledges that 5-HTR4 and PDE4D belong to the same signaling axis and follow the same molecular “modulatory” profile, which is related to cyclic nucleotides and the neurotransmitter pathway. These modulatory markers work in the same biological direction. Our results show that when these two proteins are simultaneously expressed the same way, up or down, this indicates a better prognosis. Our conclusion is that it is very important to have good balance between these proteins, in order to have optimal cAMP levels in the cells, allowing the cAMP signaling pathway to function regularly.

Studying these genes individually, we have shown that high protein expression of the *HTR4* gene was more common in the group of patients with higher total GI. This means that higher GI accompanies the activation of serotonin receptors and pathways, i.e., affects gene expression and regulation. Mutations of the *HTR4* gene can be an indicator or a consequence of GI. Liu and colleagues found a low expression level of the *HTR4* gene in both LGG and HGG, and association of high expression level of this gene and greater sensitivity of glial tumors to CTx [36].

High expression level of *PDE4* gene has been proven in brain tumors [37] and has also been proven during the progression of glial brain tumors. Consequently, it leads to increased concentrations of the PDE4D enzyme and reduction in cAMP levels, which is associated with an increased level of inflammatory cytokines such as tumor necrosis factor, interleukins 17 and 23 and interferon γ, and a decreased level of regulatory cytokines such as interleukin 10 [25]. This increase in PDE4D leads to shorter survival time and a worse outcome. All this supports our findings that synchronized expression of these proteins may be one of the key factors for longer survival and better prognosis of patients with glial brain tumors.

In addition, we analyzed the level of GI (MIN total, CIN total, total GI) on a new cohort of patients with gliomas (GBM, astrocytoma, ODG, PCA, PXA, HGAP and ependymoma of different grades (Table 1)) in order to expand our previous results, and see if GI can help better stratification of gliomas, and represent a tool for risk assessment and therapy assessment. The data about the level of MIN and CIN in CNS tumors is controversial in the literature. Tumors often have a higher MIN and a lower CIN, or vice versa. In this study, we demonstrated the presence of MIN and CIN simultaneously in all patients with brain glioma. We proved that there is a statistically significant association between MIN total and CIN total, i.e., that a higher CIN total is more prevalent in the group of patients with a higher MIN total. MIN total, CIN total and total GI clearly separate the tumor profile in terms of the degree of GI. Total GI is the most stable and statistically powerful biomarker of tumor biology in our study.

Our study did not show association between CIN total and MIN total and sex, smoking status, type, grade and category of tumor, application of CTx and RT, the protein expression status of *HTR4* and *PDE4D* genes and survival. Using Kaplan–Meier analysis, we showed that patients with brain gliomas who showed a higher level of total GI had a worse prognosis and shorter survival, but we also saw that MIN total alone, or CIN total alone did not affect survival. This indicates the fact that, total GI, consisting of MIN total and CIN total, gives a more aggressive biological profile than MIN total or CIN total alone. This suggests that total GI may be a prognostic marker of tumor aggressiveness, and a clinically relevant indicator of unfavorable disease course. It may have a role in predicting outcomes, as well as identifying high-risk patients. A direct consequence of GI are changes in the expression and regulation of various genes, which can also affect sensitivity, i.e., resistance to standard therapy. Based on this, we can conclude that total GI could play a role in therapy planning and designing therapy protocols.

The clonal evolutionary theory of the origin and progression of cancer is based on the hypothesis that with tumor progression, a decrease in the level of GI is expected, due to clonal selection. Lower level of GI could be a potential target for the detection of astrocytoma, grade 4 and their differentiation from GBM. Low GI is characteristic of GBM, IDH-wild type, while higher GI is characteristic of astrocytoma, IDH-mutant, grade 4, 3, and 2. There is also a statistically significant difference in the level of GI between astrocytomas and IDH mutant in relation to tumor grade. Astrocytoma, IDH-mutant, grade 2 is characterized by a higher level of GI compared to grade 3, while it is the lowest in grade 4. The conclusion is that during the progression of astrocytoma, IDH-mutant GI decreases [38]. Milinković et al. showed no difference in the degree of GI between anaplastic astrocytoma (according to the latest WHO classification of tumors of CNS from 2021, astrocytoma, grade 3) and primary GBM (according to the latest WHO classification of tumors of CNS from 2021, GBM, IDH-wild type). Since in the study, all analyzed GBMs were primary, which arose de novo, and not by progression from low-grade astrocytoma (secondary) (according to the latest WHO classification of tumors of CNS from 2021, astrocytoma grade 4), they also concluded that a lower level of GI could be a potential biomarker for the detection of secondary GBM [17].

## 5. Conclusions

Synchronized expression of 5-HTR4 and PDE4D proteins represents a stable modulatory signaling axis of glial tumor biology and reflects the activity of the cAMP signaling pathway associated with neurotransmission and intracellular regulation of cyclic nucleotides, but cannot independently stratify patients. They are tightly linked to each other, and represent supplementary, not primary, markers of tumor aggressiveness. We framed these proteins as part of a modulatory signaling axis, rather than independent prognostic biomarkers. They are associated with higher total GI, which could be considered a biomarker of gliomas. Moreover, our study confirms that the combination of MIN, CIN and total GI, with cluster division as the optimal approach, represents a powerful tool for biological tumor stratification, risk assessment and understanding the pathobiological spectrum of the disease. This fact opens up the possibility for the clinical application of these indicators in predicting outcomes, planning therapy and identifying patients at higher risk. Future research should validate these “biomarkers” in larger prospective cohorts.

## Figures and Tables

**Table 1:** Histopathological type of tumor, WHO grade and the total number of tumor samples.

Histopathological Type of Tumor	WHO Grade	Total Number
GBM	4	54
Astrocytoma	4	10
Astrocytoma	3	13
Astrocytoma	2	3
ODG	3	4
ODG	2	10
PCA	1	6
PXA	2	1
HGAP	3	1
Ependymoma	3	1
Ependymoma	2	1
Total		104

**Table 2:** Primer sequences, AP-PCR reaction conditions and reaction mixture.

Primer	Sequence	Low Specificity Conditions for the First 5 Cycles	High Specificity Conditions for 35 Cycles	AP-PCR Reaction Mixture
CA12F	5′-ACT GCG GCA GGA CTG AGT CT-3′	95 °C 30 s 40 °C 2′ 72 °C 1′	95 °C 30 s; 60 °C 1′ 72 °C 1′	MgCl_2_ 1.5 mM dNTP 0.6 mM primer 5 µM DNA 50 ng
CA9R	5′-CCT CCA TAG CGC CAA TGA CT-3′	95 °C 30 s 40 °C 2′ 72 °C 1′	95 °C 30 s 60 °C 1′ 72 °C 1′	MgCl_2_ 1.5 mM dNTP 0.6 mM primer 5 µM DNA 50 ng
MDR2	5′-GTT CAA ACT TCT GCT CCT GA-3′	95 °C 30 s 45 °C 2′ 72 °C 1′	95 °C 30 s 60 °C 1′ 72 °C 1′	MgCl_2_ 2.5 mM dNTP 0.4 mM primer 5 µM DNA 50 ng
C-MYC1	5′-GCT CCA AGA CGT TGT GTG TTC G-3′	94 °C 1′ 45 °C 2′ 72 °C 2′	94 °C 1′ 65 °C 1′ 72 °C 2′	MgCl_2_ 2 mM dNTP 0.6 mM primer 5.5 µM DNA 35 ng

MgCl_2_-magnesium chloride; dNTP-deoxyribonucleoside triphosphate.

**Table 3:** Percentage of positive cells (PP) and staining intensity (SI).

PP	PP Score	SI	SI Score
0%	0	No color reaction	0
1–25%	1	Weak reaction	1
26–50%	2	Moderate reaction	2
51–75%	3	Strong reaction	3
76–100%	4		

**Table 4:** Interpretation of IRS.

IRS	Interpretation
0–1	Negative
2–3	Positive: Weak
4–8	Positive: Moderate
9–12	Positive: Strong

**Table 5:** Patients’ characteristics.

Features	Number of Patients (%)
Sex	
Male	47 (45.2%)
Female	57 (54.8%)
Smoking status	
Yes	19 (18.3%)
Former	9 (8.7%)
No	76 (73.1%)
Tumor type	
Astrocytoma	26 (25.0%)
Ependymoma	2 (1.9%)
CAG	8 (7.7%)
GBM	54 (51.9%)
ODG	14 (13.5%)
Tumor category	
LGG	21(20.2%)
HGG	83(79.8%)
Tumor grade	
I	6 (5.8%)
II	15 (14.4%)
II	19 (18.3%)
IV	64 (61.5%)
RT	
No	91 (87.5%)
Yes	13 (12.5%)
CTx	
No	96 (92.3%)
Yes	8 (7.7%)
HTR4	
Low	87 (83.7%)
High	17 (16.3%)
PDE4D	
Low	89 (85.6%)
High	15 (14.4%)

**Table 6:** Association between 5-HTR4 and clinical, histopathological parameters, PDE4D protein expression and status (event vs. censored).

		HTR4			
			Low	High	
		Number of Patients (%)			*p* Value
Sex	Male	47 (45.2%)	39 (44.8%)	8 (47.1%)	0.866
	Female	57 (54.8%)	48 (55.2%)	9 (52.9%)	
Smoking	Yes	19 (18.3%)	17 (19.5%)	2 (11.8%)	0.641
	Former	9 (8.7%)	8 (9.2%)	1 (5.9%)	
	No	76 (73.1%)	62 (71.3%)	14 (82.4%)	
Type of tumor	Astrocytoma	26 (25.0%)	22 (25.3%)	4 (23.5%)	0.754
	Ependymoma	2 (1.9%)	2 (2.3%)	0 (0.0%)	
	CAG	8 (7.7%)	6 (6.9%)	2 (11.8%)	
	GBM	54 (51.9%)	44 (50.6%)	10 (58.8%)	
	ODG	14 (13.5)	13 (14.9%)	1 (5.9%)	
Tumor cathegory	LGG	21 (20.2%)	18 (20.7%)	3 (17.6%)	0.775
	HGG	83 (79.8%)	69 (79.3%)	14 (82.4%)	
Grade	I	6 (5.8%)	5 (5.7%)	1 (5.9%)	0.987
	II	15 (14.4%)	13 (14.9%)	2 (11.8%)	
	III	19 (18.3%)	16 (18.4%)	3 (17.6%)	
	IV	64 (61.5%)	53 (60.9%)	11 (64.7%)	
RT	No	91 (87.5%)	77 (88.5%)	14 (82.4%)	0.483
	Yes	13 (12.5%)	10 (11.5%)	3 (17.6%)	
CTx	No	96 (92.3%)	81 (93.1%)	15 (88.2%)	0.491
	Yes	8 (7.7%)	6 (6.9%)	2 (11.8%)	
PDE4D	Low	89 (85.6%)	78 (89.7%)	11 (64.7%)	0.007 OR = 4.727
	High	15 (14.4%)	9 (10.3%)	6 (35.3%)	
STATUS	Censored	34 (32.7%)	29 (33.3%)	5 (29.4%)	0.753
	Event	70 (67.3%)	58 (66.7%)	12 (70.6%)	

**Table 7:** Association between MIN total (mean, cluster and median) and CIN total (mean, cluster and median).

		CIN Total Cluster 0.165			CIN Total Mean 0.17619			CIN Total Median 0.16810		
		Lower	Upper	*p* Value	Lower	Upper	*p* Value	Lower	Upper	*p* Value
MIN total cluster	Lower	34 (56.7%)	26 (43.3%)		38 (63.3%)	22 (36.7%)		35 (58.3%)	25 (41.7%)	
0.179	Upper	17 (38.6%)	27 (61.4%)	0.069	17 (38.6%)	27 (61.4%)	0.013 OR = 2.743 χ^2^ = 6.214	17 (38.6%)	27 (61.4%)	0.047 OR = 2.224 χ^2^ = 3.939
MIN total mean	Lower	34 (56.7%)	26 (43.3%)		38 (63.3%)	22 (36.7%)		35 (58.3%)	25 (41.7%)	
0.18057	Upper	17 (38.6%)	27 (61.4%)	0.069	17 (38.6%)	27 (61.4%)	0.013 OR = 2.743 χ^2^ = 6.214	17 (38.6%)	27 (61.4%)	0.047 OR = 2.224 χ^2^ = 3.939
MIN total median	Lower	31 (60.8%)	21 (39.6%)		34 (65.4%)	18 (34.6%)		32 (61.5%)	20 (38.5%)	
0.15776	Upper	20 (39.2%)	32 (60.4%)	0.031 OR = 2.362 χ^2^ = 4.656	21 (40.4%)	31 (59.6%)	0.011 OR = 2.788 χ^2^ = 6.522	20 (38.5%)	32 (61.5%)	0.019 OR = 2.560 χ^2^ = 5.538

**Table 8:** Association between total GI (mean, cluster and median) and sex, smoking status, type, grade and category of tumor, application of CTx and RT, protein expression status of *HTR4* and *PDE4D* genes and status (event vs. censored).

						Total GI					
			Cluster 0.314			Mean 0.35676			Median 0.34675		
			Lower	Upper		Lower	Upper		Lower	Upper	
		Number of Patients (%)			*p* Value			*p* Value			*p* Value
Sex	Male	47 (45.2)	24 (57.1)	23 (37.1)	0.044 OR = 2.261	30 (53.6)	17 (35.4)	0.064	28 (53.8)	19 (36.5)	0.076
	Female	57 (54.8)	18 (42.9)	39 (62.9)		26 (46.4)	31 (64.6)		24 (46.2)	33 (63.5)	
					χ^2^ = 4.062						
Smoking	Yes	19 (18.3)	8 (19.0)	11 (17.7)	0.898	10 (17.9)	9 (18.8)	0.822	9 (17.3)	10 (19.2)	0.897
	Former	9 (8.7)	3 (7.1)	6 (9.7)		4 (7.1)	5 (10.4)		4 (7.7)	5 (9.6)	
	No	76 (73.1)	31 (73.8)	45 (72.6)		42 (75)	34 (70.8)		39 (75.0)	37 (71.2)	
Type of tumor	Astrocytoma	26 (25.0)	13 (31.0)	13 (21.0)	0.349	16 (28.6)	10 (20.8)	0.073	14 (26.9)	12 (23.1)	0.421
	Ependymoma	2 (1.9)	1 (2.4)	1 (1.6)		2 (3.6)	0 (0.0)		1 (1.9)	1 (1.9)	
	CAG	8 (7.7)	1 (2.4)	7 (11.3)		3 (5.4)	5 (10.4)		3 (5.8)	5 (9.6)	
	GBM	54 (51.9)	20 (47.6)	34 (54.8)		24 (42.9)	30 (62.5)		24 (46.2)	30 (57.7)	
	ODG	14 (13.5)	7 (16.7)	7 (11.3)		11 (19.6)	3 (6.3)		10 (19.2)	4 (7.7)	
Tumor category	LGG	21 (20.2)	6 (14.3)	15 (24.2)	0.217	12 (21.4)	9 (18.8)	0.734	10 (19.2)	11 (21.2)	0.807
	HGG	83 (79.8)	36 (85.7)	47 (75.8)		44 (78.6)	39 (81.3)		42 (80.8)	41 (78.8)	
Tumor Grade	I	6 (5.8)	1 (2.4)	5 (8.1)	0.11	2 (3.6)	4 (8.3)	0.099	2 (3.8)	4 (7.7)	0.275
	II	15 (14.4)	5 (11.9)	10 (16.1)		10 (17.9)	5 (10.4)		8 (15.4)	7 (13.5)	
	III	19 (18.3)	12 (28.6)	7 (11.3)		14 (25)	5 (10.4)		13 (25.0)	6 (11.5)	
	IV	64 (61.5)	24 (57.1)	40 (64.5)		30 (53.6)	34 (70.8)		29 (55.8)	35 (67.3)	
RT	No	91 (87.5)	36 (85.7)	55 (88.7)	0.650	47 (83.9)	44 (91.7)	0.234	44 (84.6)	47 (90.4)	0.374
	Yes	13 (12.5)	6 (14.3)	7 (11.3)		9 (16.1)	4 (8.3)		8 (15.4)	5 (9.6)	
CTx	No	96 (92.3)	39 (92.9)	57 (91.9)	0.863	50 (89.3)	46 (95.8)	0.212	47 (90.4)	49 (94.2)	0.462
	Yes	8 (7.7)	3 (7.1)	5 (8.1)		6 (10.7)	2 (4.2)		5 (9.6)	3 (5.8)	
HTR4	Low	87 (83.7)	39 (92.9)	48 (77.4)	0.037 OR = 3.792	50 (89.3)	37 (77.1)	0.093	47 (90.4)	40 (76.9)	0.063
	High	17 (16.3)	3 (7.1)	14 (22.6)		6 (10.7)	11 (22.9)		5 (9.6)	12 (23.1)	
PDE4D	Low	89 (85.6)	38 (90.5)	51 (82.3)	0.242	51 (91.1)	38 (79.2)	0.085	47 (90.4)	42‚1 (80.8)	0.163
	High	15 (14.4)	4 (9.5)	11 (17.7)		5 (8.9)	10 (20.8)		5 (9.6)	10 (19.2)	
STATUS	Censored	34 (32.7)	16 (38.1)	18 (29.0)	0.334	23 (41.1)	11 (22.9)	0.049 OR = 2.344	21 (40.4)	13 (25.0)	0.094
	Event	70 (67.3)	26 (61.9)	44 (71.0)		33 (58.9)	37 (77.1)		31 (59.6)	39 (75.0)	
								χ^2^ = 3.871			

## Data Availability

The data presented in this study are available on request from the corresponding author.

## Abbreviations

The following abbreviations are used in this manuscript:

CNS	central nervous system
GBM	glioblastoma
LGG	low-grade glioma
IHC	immunohistochemical
GI	genomic instability
MIN, MSI	microsatellite instability
CIN	chromosomal instability
WHO	World Health Organization
IDH	isocitrate dehydrogenase
ODG	oligodendroglioma
HGG	high-grade glioma
CAG	circumscribed astrocytic glioma
PCA	pilocytic astrocytoma
HGAP	high-grade astrocytoma with piloid features
PXA	pleomorphic xanthoastrocytoma
MN1	MN1 proto-oncogene, transcriptional regulator
DNA	deoxyribonucleic acid
CTx	chemotherapy
RT	radiotherapy
SNI	single nucleotide instability
MMR	mismatch repair
AP-PCR	arbitrarily primed polymerase chain reaction
5-HTR	5-hydroxytryptamine (serotonine) receptor
AC	adenylyl cyclase
ATP	adenosine triphosphate
cAMP	3′-5′-cyclic adenosine monophosphate
PKA	protein kinase A
EPAC	exchange protein activated by cAMP
CREB	CRE-binding protein
CREM	cAMP-responsive modulator
ATF1	activating transcription factor 1
Rap1	Ras-associated protein 1
Rap2	Ras-associated protein 2
5-HTR4	5-hydroxytryptamine (serotonine) receptor 4
TSG	tumor-suppressor gene
PDE	phosphodiesterase
PDE4D	cAMP-specific 3′,5′-cyclic phosphodiesterase 4D
CA12 F	carbonic anhydrase 12 forward
CA 9 R	carbonic anhydrase 9 reverse
MDR2	multi-drug resistance 2
C-MYC1	cellular myelocytomatosis oncogene 1
MgCl_2_	magnesium chloride
dNTP	deoxyribonucleoside triphosphate
NTC	no-template control
FEPE	formalin-fixed paraffin-embedded
DAB	diaminobenzidine
IRS	immunoreactive score
SI	staining intensity
PP	percentage of positive cells
OR	odds ratio
